# Nectar Sugar Production across Floral Phases in the *Gynodioecious Protandrous* Plant *Geranium sylvaticum*

**DOI:** 10.1371/journal.pone.0062575

**Published:** 2013-04-22

**Authors:** Sandra Varga, Carolin Nuortila, Minna-Maarit Kytöviita

**Affiliations:** 1 Department of Biological and Environmental Science, University of Jyväskylä, Jyväskylä, Finland; 2 Tuulimyllynkatu, Oulu, Finland; Centro de Investigación y de Estudios Avanzados, Mexico

## Abstract

Many zoophilous plants attract their pollinators by offering nectar as a reward. In gynodioecious plants (i.e. populations are composed of female and hermaphrodite individuals) nectar production has been repeatedly reported to be larger in hermaphrodite compared to female flowers even though nectar production across the different floral phases in dichogamous plants (i.e. plants with time separation of pollen dispersal and stigma receptivity) has rarely been examined. In this study, sugar production in nectar standing crop and secretion rate were investigated in *Geranium sylvaticum*, a gynodioecious plant species with protandry (i.e. with hermaphrodite flowers releasing their pollen before the stigma is receptive). We found that flowers from hermaphrodites produced more nectar than female flowers in terms of total nectar sugar content. In addition, differences in nectar production among floral phases were found in hermaphrodite flowers but not in female flowers. In hermaphrodite flowers, maximum sugar content coincided with pollen presentation and declined slightly towards the female phase, indicating nectar reabsorption, whereas in female flowers sugar content did not differ between the floral phases. These differences in floral reward are discussed in relation to visitation patterns by pollinators and seed production in this species.

## Introduction

Most flowering plants rely on insects to effectively transfer pollen and get ovules fertilised. Selection has led to the use of various means that help to ensure repeated visitation by pollinating animals. One mechanism to attract pollinators is to offer rewards, the most important ones being pollen and nectar [1], heat [2] and shelter [3]. Nectar is a sugar-rich aqueous solution with 10% to 75% of sugars [4] involving various proportions of sucrose, fructose and glucose in the vast majority of plants analysed [5]. The role of nectar in mediating the interaction between plants and their pollinators is pivotal: nectar is used as an energy source for pollinators and therefore is subject to selection pressures by pollinators. Thus, the amount of nectar reward is positively correlated with the number of pollinator visits (e.g. [6]), the number of flowers visited within a plant [7] and the duration of the visit within a flower [8]. Flowers must provide enough nectar to attract pollinators; however, they must also limit this reward so that pollinators will go on to visit other flowers [9]. Other important constituents of nectar are amino acids, alkaloids, antioxidants, vitamins and lipids [10]. The function of these secondary compounds may include selecting for the right pollinator, deter antagonists, or even regulate the duration of pollinator visits (e.g. [11], [12]).

Many variables act on determining the amount of nectar available in a flower (see [13], and references there). Nectar secretion rate can change during the day [14], during flower life span [15] and may differ among flowering seasons and years [16]. Furthermore, the biotic environment may affect nectar production, as for example herbivory has been shown to have contrasting effects on nectar production ([17], and references there). Moreover, there is large variation in nectar volume due to environmental conditions including light [18], water [19], nutrients [20], temperature [21] and CO_2_ concentration [22]. Secretion rate can also vary greatly among flowers within plants (e.g. [23]–[25]). Last, but not least, nectar production may depend on the gender of a flower in sexually dimorphic plants (reviewed in [26]).

Theory predicts that in sexually dimorphic plants, where the sexual functions are separated in different individuals, the gender in which fitness is more limited by pollinators should be selected to produce the greater reward [27]. It is well established that female flowers in gynodioecious species (i.e. where populations are composed of female and hermaphrodite individuals) are usually smaller (reviewed in [28]) and that they produce less nectar than hermaphrodite flowers (Table 1). However, according to our knowledge, few studies have measured nectar production among the different floral phases in gynodioecious plants (but see [29], [30]). In the present work, we studied nectar production in the gynodioecious species *Geranium sylvaticum* during different floral phases. In addition to having sexually dimorphic flowers, the hermaphrodite flowers of *G. sylvaticum* exhibit dichogamy, i.e. time separation of pollen dispersal and stigma receptivity. Nectar production in the two sexes of *G. sylvaticum* is unknown and given the important implication of nectar production on insect visitation and on plant resource allocation patterns, elucidating the role of nectar production in the two sexes is necessary to fully understand how gynodioecy functions in this species. Thus, the aims of this study were (1) to compare pollinator rewards in terms of nectar sugar production between female and hermaphrodite *G. sylvaticum* flowers and (2) to determine sugar production among floral stages in both genders.

**Table 1:** Studies reporting nectar production in gynodioecious plants.

Plant species	Method	Nectar	Perianth size	Reference
*Dianthus sylvestris*	Field, SR	H>F 1, 2	H>F	[46]
		H = F ^3^		[46]
*Echium vulgare*		H>F 1	H>F	[54], [61]
*Fragaria virginiana*	Greenhouse, SR	H>F ^2^	H>F	[62]
*Fuchsia excorticata*	SR	H>F 1	H>F	[29]
*Fuchsia lycioides*	Field, SC	H>F 1	H>F	[52]
*Geranium sylvaticum*	Field, SR and SC	H>F ^2^	H>F	[34], this study
*Glechoma longituba*	Field, SR	H>F 1	H>F	[51]
		H = F ^3^		[51]
*Hebe stricta*	SR and SC	H>F 1 SR	H = F	[63]
		H = F 1 SC		[63]
*Iris douglasiana*		H>F 1, ^2^	H>F	[64]
*Lobelia spicata*	Field	H>F 1	H>F	[65]
*Opuntia quimilo*	Field	H>F ^3^	H<F	[66]
*Phacelia linearis*		H = F 1, 2	H>F	[67], [68]
*Prunus mahaleb*		H>F 1	H = F	[69]
*Sidalcea oregana*	Greenhouse, SR	H>F ^2^	H>F	[70]
*Silene stockenii*		H>F 1, 3	H>F	[30]
*Silene vulgaris*	Common garden, SR	H>F ^2^	H>F	[71]

SR  =  Secretion rate in 24 h, SC  =  standing crop, H =  hermaphrodite flower, F =  female flower.

1 volume; ^2^sugar content; ^3^sugar concentration.

H>F, the value for the particular trait was higher in hermaphrodite than female flower. H<F, the value for the particular trait was higher in female than hermaphrodite flower. H = F, there was no statistical difference for the particular trait between female and hermaphrodite flowers.

## Materials and Methods

### Study species and flower characters

**Figure 1:**
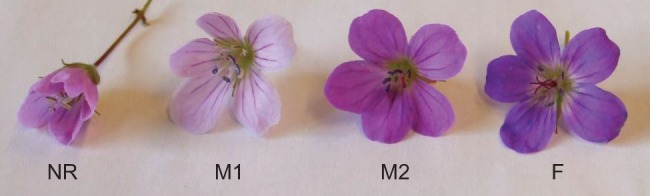
The floral phases in hermaphrodite *G. sylvaticum* flowers. NR  =  Non-receptive phase, MI  =  Male I phase, MII  =  Male II phase, F =  Female phase. See Materials and methods for more details. Flowers were collected from different plant individuals, and therefore show differences in coloration.

*Geranium sylvaticum* is a self-compatible, perennial plant with Eurasian distribution [31], found in herb-rich forests, meadows and along roads. Most populations in Finland and Russia are gynodioecious [32], [33], with female plants bearing flowers with rudimentary stamens and no pollen, and hermaphrodite plants bearing protandrous flowers (i.e. hermaphrodite flowers that release their pollen before the stigma is receptive) with one to ten functional stamens divided in two whorls of five and producing viable pollen. Both female flowers and hermaphrodite flowers produce a fixed number of ten ovules per flower, but female plants produce more seeds than hermaphrodites [34], [35] even though there is some variation among populations and years [32]. Hermaphrodite flowers are larger than female flowers [34]. In hermaphrodite flowers, following bud opening, petals start unfolding and the stamens and the pollen sacs become visible (Fig. 1; non-receptive phase, referred as NR hereafter). This phase may last from 30 minutes to often four hours or longer, strongly depending on the weather conditions (S. Varga, unpublished data). After this phase, the inner whorl of the stamens dehisces (Fig. 1; male I phase, referred as M1 hereafter) exposing the pollen and this is followed by the dehiscence of the outer whorl of stamens (Fig. 1; male II phase, referred as M2 hereafter). These phases may last from 30 minutes to often six hours or longer. Until this point the five stigmatic lobes remain closely joined to each other and are not receptive. Usually, after a maximum of 24 hours after bud opening, the stigma lobes start unfolding and expose the five papillate stigmatic surfaces becoming receptive for pollen (Fig. 1; female phase, referred as F hereafter). Flowers remain in the female phase between 1.5 hours to often six hours or even longer before the stigma lobes close again and the petals shrivel and drop. In female flowers, following bud opening, petals start unfolding for one to often five hours or even longer (NR phase) and then the stigma unfolds becoming receptive for pollen (F phase) which may last from two to often more than six hours or even longer (strongly depending again on the weather conditions).

**Figure 2:**
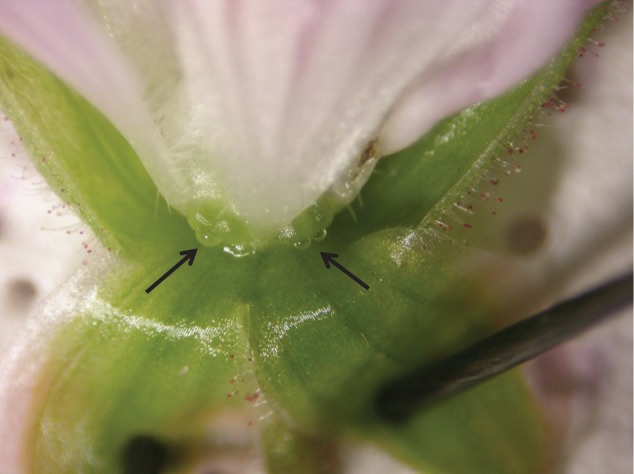
Position of the nectaries in a *G. sylvaticum* flower. Arrows show nectar droplets.

Therefore, in female flowers only two phases can be recognised: NR and F. On average, both female and hermaphrodite flowers remain open up to two to three days (S. Varga and CD. Soulsbury, unpublished data) even though the exact duration is strongly related to temperature and insect visitation rates. Nectar is produced in nectaries at the base of the flowers (Fig. 2). In the field, *G. sylvaticum* starts flowering in mid-June, the plants are pollinated by bumblebees, syrphid flies and other dipterans [36]. Previous studies have shown that hermaphrodite flowers of *G. sylvaticum* receive more visits by pollinating insects in general [37], while bumblebees visit both genders equally often [36].

### Nectar sampling

Samples of floral nectar were collected during the peak of flowering in July 2008 (5^th^ July – 18^th^ July) from a *G. sylvaticum* population naturally growing at Oulu University Botanical Gardens (65°03′N, 25°27′E). Pollinators of *G. sylvaticum* are reported to be most active between 10.00 h and 14.00 h [37] and therefore samples were collected at two different times, at the beginning of the peak of maximum activity (11.00 h, referred as ‘Morning’ hereafter), and after the maximum activity (15.00 h, referred as ‘Afternoon’ hereafter). Each day, we randomly selected flowers from different plants growing within the population. Part of the selected flowers were protected from pollinator visits using mesh bags (referred as ‘Bagged’ flowers hereafter) for 24 h before nectar was extracted to estimate sugar accumulation in 24 hours. Alternatively, part of the selected flowers was left available for pollinators (referred as ‘Open’ flowers hereafter) to estimate nectar standing crop. On each sampling occasion, Bagged and Open flowers with different floral phases were chosen from different plants to control collecting samples from all phases, genders and times across the different sampling days. We aimed at obtaining between 15 and 20 samples from each floral phase, gender and time of the day for both Bagged and Open flowers. However, it was not always possible to find all combinations and the number of samples from each combination ranged from 3–46 samples (with an average of 21 samples per combination), giving a final sample sizes of 297 for Open flowers and 196 for Bagged flowers. The lowest replication numbers were collected in afternoon samples from Female flowers in NR phase (Bagged and Open) since it was not possible to find female flowers in such a phase. The population was composed of more than 100 plants, but it is possible that flowers from the same individual plant could have been harvested on different days. It was not possible to reliably extract nectar from the flowers using microcapillars, and therefore, no information on volume and nectar concentration could be attained. Flowers were cut from the plant, placed with the peduncle into water in an Eppendorf tube to minimise the risk of desiccation, and brought to the lab within 30 min from the time of cutting. We noted flower gender (female, hermaphrodite) and the floral sexual phase. Nectar was extracted with paper wicks as described in [38] under a stereomicroscope to calculate total carbohydrate content. Nectar samples were then kept in an exiccator until total carbohydrate content was determined using the anthrone method [10], pp: 176–177). We prepared a series of sugar standards ranging from 0 to 50 µg of total sugar per mL of standard using equal amounts of fructose and glucose because even though nectar composition is unknown for *G. sylvaticum*, in other closely related *Geranium* species similar dominant proportions of fructose and glucose have been reported [5].

Sugars in the paper wicks were redissolved by vortexing the wicks for 1 minute in 5 mL boiling distilled water. The reagent blank, the sugar standards and 2 mL of the sample solution were placed into test tubes in an ice bath. Then 4 mL of anthrone reagent (0.4 g anthrone in 200 mL concentrated sulphuric acid) was added into each tube. Tubes were vortexed shortly and then placed in a boiling water bath for 10 minutes. The absorbance was read with a BioSpec-1601E spectrophotometer (Shimadzu, Kyoto, Japan) at 620 nm after allowing the tubes to cool down for 20 minutes.

Air temperature and humidity at the time of the samplings were obtained from the Finnish Meteorological Institute (http://en.ilmatieteenlaitos.fi).

### Statistical analyses

To infer differences in nectar accumulation between plant gender (Female, Hermaphrodite), sampling time (Morning, Afternoon) and floral phase (Non-receptive, Male I, Male II, Female) which was nested within gender, a three-way ANOVA with Tukey's *post-hoc* comparisons was used. Nectar accumulation was log-transformed to meet ANOVA assumptions and data were analysed separately for open and bagged flowers in order to differentiate standing crop and total sugar content. Air temperature and humidity at the time of sampling were included as covariates in the models. Analyses were performed using PASW v.18 (SPSS, Chicago, Illinois, USA).

## Results

**Figure 3:**
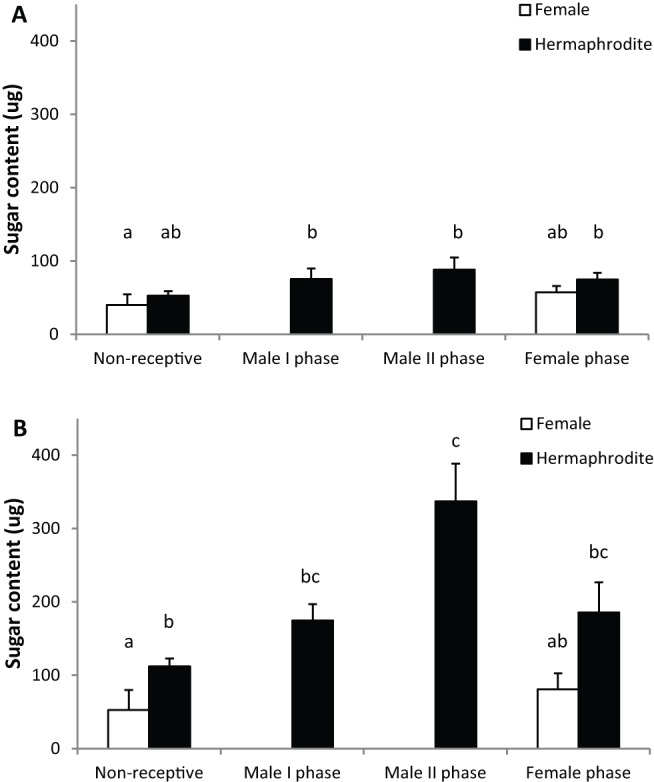
Sugar content in nectar standing crop and nectar accumulated in 24 hours. (A) Sugar content (μg per flower) nectar standing crop and (B) nectar accumulated in 24 hours in female (white bars) and hermaphrodite (black bars) *Geranium sylvaticum* flowers across the different floral phases. Bars indicate mean + S.E. Different letters above the bars indicate statistically significant differences among groups at *P*<0.05.

Both nectar measurements were statistically affected by air temperature and humidity at the time of sampling (Table 2). Total carbohydrate per flower ranged between 0–1120 μg (average 103.3±6.2 μg). Nectar standing crop sugar content (measured from open flowers) was 2.5 times smaller than accumulated 24 h nectar measured from Bagged flowers, suggesting that flowers were visited by pollinators (Fig. 3). The standing crop differences were small between the genders but statistically significant (Table 2; Fig. 3a) and hermaphrodite flowers had slightly larger standing crop sugar content than female flowers. Significant variation was also observed among floral phases, with the highest standing crop sugar content in hermaphrodite flowers in male phases and the lowest in female flowers in NR phase (Table 2; Fig. 3a). We did not detect any statistically significant differences in sugar content in nectar standing crop between samples taken in the morning or in the afternoon and there was no significant interaction between time and gender (Table 2).

**Table 2:** Statistical results of the ANOVA models for nectar standing crop (N = 297 flowers) and nectar accumulated in 24 hours (N = 196 flowers).

	Nectar standing crop (Open flowers)			Nectar accumulation (Bagged flowers)		
	*df*	F	*P*	*df*	F	*P*
Temperature	1	37.055	**<0.01**	1	50.276	**<0.01**
Humidity	1	3.619	0.06	1	4.849	**0.03**
Time	1	0.294	0.59	1	1.760	0.19
Gender	1	14.357	**<0.01**	1	24.807	**<0.01**
Phase (Gender)	4	2.368	**0.05**	4	3.430	**0.01**
Time * Gender	1	0.007	0.94	1	1.227	0.27
Error	208			116		

Analyses were performed on log-transformed data. Significant effects are shown in bold.

The genders and the different flower phases showed significant differences in nectar sugar accumulation in 24 h regardless of the sampling time (Table 2; Fig. 3b). In females, even though sugar content was slightly larger in F compared to NR phase, the difference was not statistically significant. Hermaphrodite flowers contained the largest sugar content when the second whorl of stamens was presenting the pollen (MII phase; Fig. 3b) and the sugar content decreased towards the female phase, possibly indicating unconsumed nectar reabsorption.

## Discussion

### Nectar sugar production in female and hermaphrodite G. sylvaticum flowers

According to our knowledge, differences in nectar production in gynodioecious species have only been described in 16 species, using various methods and reporting different parameters (Table 1). In agreement with most of these studies, we observed that hermaphrodite flowers of *G. sylvaticum* produced more sugar than flowers from females. Several, not mutually exclusive explanations have been proposed to explain higher nectar reward in hermaphrodite flowers over female ones (reviewed in [39]). First, Bateman's principle [27] and the male function hypothesis predict that sexual selection should predominantly act on floral traits through the male function of flowers, since male fecundity is generally limited by pollinator visits and female fecundity by nutrient limitation. However, seed production in *G. sylvaticum* is limited by both pollen and resource availability in both genders [40], which is not in line with Bateman's principle. Second, differences between the genders in nectar production may reflect the relatively higher cost of reproduction in females compared to hermaphrodites ([28], [41]). Nectar is energetically expensive: it may use up to 35% of a plant's available carbon [42], and it may entail a cost for the plant in terms of reduced growth and/or reproduction [43]. In *G. sylvaticum*, when differences in seed output are reported, females produce more seeds than hermaphrodites ([34], [35]) even though there is some variation among populations and years [32] and therefore, differences in sugar content between genders could reflect this different resource investment. However, also pollen production represents a significant resource sink for plants [44], and theoretically, females may allocate resources not invested in pollen to produce nectar. Third, nectar production is strongly positively correlated with perianth size (e.g. [45]) and therefore, since hermaphrodite flowers are larger than females (Table 1), they are also expected to produce more nectar. Finally, females when producing less nectar might be less attractive than hermaphrodites to floral enemies, thus minimising or escaping attacks by herbivores and pathogens [46]. Taken together, nectar production patterns in *G. sylvaticum* might have evolved as a response to both predation pressure by flower enemies and pollinators.

### Nectar sugar production among floral stages

Nectar sugar accumulation was maximal during the Male II phase and slightly decreased towards the female phase, indicating sugar reabsorption in hermaphrodites. However, the nectar sugar standing crop measurements showed rather uniform sugar content across the different flower phases and were lower only in the non-receptive phase in the female flowers. In addition, floral phase affected nectar accumulation significantly in hermaphrodite flowers, but not in female flowers. Overall, it is somehow unexpected that nectar was offered already in flowers in the non-receptive phase when ovules cannot be fertilised nor pollen can be removed from the flowers by pollinators. It seems unreasonable to produce nectar at this point, unless early nectar production is used to “advertise” the flowers for later on, or alternatively, flowers have no mechanism to avoid nectar secretion at this point. The latter point is supported by copious literature suggesting that in most plants flowers begin to secrete nectar before pollinators could pollinate and in some cases even before the flowers open ([47], and references therein). In addition, pollinators visiting non-receptive flowers might deposit pollen on the stigma, so when the stigma becomes receptive the pollen, if still viable, could fertilize the ovules, as suggested for *Cerinthe major* [48].

### Nectar, insect visits and seed production

There was a larger difference between nectar standing crop and nectar accumulation in the hermaphrodite compared to the female flowers. This may indicate that hermaphrodite flowers received more frequent insect visits. Nectar accumulation showed that the females rewarded the visitors with less sugar. In this study, we did not monitor insect visitation rates, but when floral visitors were monitored altogether, preferences for hermaphrodites over females in *Geranium* have been documented previously in the field ([37], [49]). When inspecting bumblebees separately from other visiting insects, Varga and Kytöviita [36] observed no difference in the visitation frequency with respect to the genders. Why do bumblebees not favour hermaphrodite *G. sylvaticum*? Bumblebees may show floral constancy ([4]; and references there) and the relatively low frequency of female flowers in *G. sylvaticum* populations [32] may prevent the bumblebees from discriminating the female flowers. Information about the correlation between floral visits and floral phase is lacking for this species and thus the implications of our finding cannot be fully evaluated. Nevertheless, differences in the amount of nectar reward produced have been shown to affect the pattern of visitation by pollinators (e.g. [50]). Studies on gynodioecious plant species have shown that hermaphrodites are usually more often visited than females by pollinators and the reasons for this preference have been attributed to the larger floral size or total floral display (e.g. [51]) and/or the presence of more nectar (e.g. [29]) or pollen (e.g. [52]) rewards in hermaphrodites. Within hermaphrodite flowers, the greater number of visits during the male phase, the better the reproductive success of these flowers in terms of pollen exported. Indeed, in several protandrous species, nectar production is higher in the male phase than in the female phase ([53]–[57]), and pollinators have been found to prefer visiting flowers during the relatively more rewarding male phase ([53], [54], [58]–[60]). In gynodioecious plants, we are aware of only two studies reporting nectar production across the different floral phases: Talavera *et al.* [30] reported higher nectar production during the female phase of hermaphrodite flowers in *Silene stockenii* whereas Delph and Lively [29] found that nectar production in hermaphrodite flowers of *Fuchsia excorticata* peaked just after dehiscence of the anthers, the time when a visit by a pollinator would be most likely to result in pollen removal from the flowers.

### Conclusions

Even though we only measured temporal patterns of nectar secretion, our results show that female *G. sylvaticum* flowers offer less sugar to pollinators than hermaphrodite flowers. Lower sugar content in females could free resources for seed production and may also reduce antagonistic visits. As nectar is costly to produce and as seed production has been shown to be partially resource limited in *G. sylvaticum*, this may, at least partially, explain why females are able to produce more seeds than hermaphrodites in this species. Whether this is the case and why bumblebees do not discriminate the genders remain open questions. Furthermore, the relationship between insect visitation pattern and floral phase need to be elucidated. Many factors determine the amount of nectar available for pollinators. Clearly, more studies are needed to understand how pollinator rewards are linked with mating success and resource trade-offs in this gynodioecious species. Ideally, a systemic sampling of all flowers per plant through the entire flower life span should be conducted.
