# Correction: Nectar Sugar Production across Floral Phases in the *Gynodioecious Protandrous* Plant *Geranium sylvaticum*

**DOI:** 10.1371/annotation/5249fbd5-091a-41c3-8b58-41cf01c5c7c6

**Published:** 2013-08-16

**Authors:** Sandra Varga, Carolin Nuortila, Minna-Maarit Kytöviita

The words "Gynodioecious" and "Protandrous" in the article title were incorrectly italicized. The correct title is: Nectar Sugar Production across Floral Phases in the Gynodioecious Protandrous Plant *Geranium sylvaticum*. The correct citation is: Varga S, Nuortila C, Kytöviita M-M (2013) Nectar Sugar Production across Floral Phases in the Gynodioecious Protandrous Plant *Geranium sylvaticum*. PLoS ONE 8(4): e62575. doi:10.1371/journal.pone.0062575. 

